# GLORY: Generator of the Structures of Likely Cytochrome P450 Metabolites Based on Predicted Sites of Metabolism

**DOI:** 10.3389/fchem.2019.00402

**Published:** 2019-06-12

**Authors:** Christina de Bruyn Kops, Conrad Stork, Martin Šícho, Nikolay Kochev, Daniel Svozil, Nina Jeliazkova, Johannes Kirchmair

**Affiliations:** ^1^Department of Computer Science, Center for Bioinformatics (ZBH), Faculty of Mathematics, Informatics and Natural Sciences, Universität Hamburg, Hamburg, Germany; ^2^CZ-OPENSCREEN: National Infrastructure for Chemical Biology, Department of Informatics and Chemistry, Faculty of Chemical Technology, University of Chemistry and Technology Prague, Prague, Czechia; ^3^Ideaconsult Ltd., Sofia, Bulgaria; ^4^Department of Analytical Chemistry and Computer Chemistry, University of Plovdiv, Plovdiv, Bulgaria; ^5^Department of Chemistry, University of Bergen, Bergen, Norway; ^6^Computational Biology Unit (CBU), University of Bergen, Bergen, Norway

**Keywords:** metabolism prediction, metabolite structure prediction, rule-based approach, sites of metabolism, xenobiotic metabolism, cytochrome P450, metabolites

## Abstract

Computational prediction of xenobiotic metabolism can provide valuable information to guide the development of drugs, cosmetics, agrochemicals, and other chemical entities. We have previously developed FAME 2, an effective tool for predicting sites of metabolism (SoMs). In this work, we focus on the prediction of the chemical structures of metabolites, in particular metabolites of xenobiotics. To this end, we have developed a new tool, GLORY, which combines SoM prediction with FAME 2 and a new collection of rules for metabolic reactions mediated by the cytochrome P450 enzyme family. GLORY has two modes: MaxEfficiency and MaxCoverage. For MaxEfficiency mode, the use of predicted SoMs to restrict the locations in the molecule at which the reaction rules could be applied was explored. For MaxCoverage mode, the predicted SoM probabilities were instead used to develop a new scoring approach for the predicted metabolites. With this scoring approach, GLORY achieves a recall of 0.83 and can predict at least one known metabolite within the top three ranked positions for 76% of the molecules of a new, manually curated test set. GLORY is freely available as a web server at https://acm.zbh.uni-hamburg.de/glory/, and the datasets and reaction rules are provided in the Supplementary Material.

## Introduction

Metabolism is responsible for creating metabolites with different physicochemical and pharmacological properties compared to those of the original parent molecule. Xenobiotic metabolism in particular is directly relevant for humans, especially as it relates to, for example, the development of drugs, cosmetics, and agrochemicals. In fact, it is supposed that metabolism is the main clearance pathway for the vast majority of all xenobiotics (Kirchmair et al., 2015). However, metabolism can also result in pharmacologically active metabolites as well as toxic metabolites (Testa et al., 2012).

The cytochrome P450 (CYP) family of enzymes plays an important role in the metabolism of xenobiotics, especially in the formation of first-generation metabolites, of which roughly 60% are formed by CYPs (Testa et al., 2012). The importance of CYPs to drug discovery is clear from the observation that many drugs are metabolized by CYPs; common estimates range from 50% (Di, 2014) to 80% (Testa et al., 2012). A detailed meta-analysis of the metabolites of over 1,000 different xenobiotic substrates carried out by Testa et al., showed that 40% of all metabolites are formed by CYPs, including a substantial proportion of all toxic or highly reactive metabolites (Testa et al., 2012).

There are 57 known human CYP enzymes, the majority of which are primarily involved in endogenous metabolism. The CYP2 and CYP3 subfamilies are mainly responsible for metabolizing xenobiotics (Testa et al., 2012), and the key CYP isozymes for drug metabolism are CYP3A4, 3A5, 2D6, 2C8, 2C9, 2C19, 1A1, 2B6, and 2E1 (Di, 2014). Among the xenobiotic-metabolizing CYP isozymes, the binding pockets vary greatly; in some cases the binding pocket of a single isozyme is highly flexible and can accommodate a broad range of substrates with widely varying sizes (Kirchmair et al., 2015).

Computational methods can make a significant contribution to predicting xenobiotic metabolism, because they can be used to quickly make predictions that can focus the experimental aspects of the drug development process. Such a focusing effect is both cost-effective and time-effective (Kirchmair et al., 2015).

One relatively well-developed aspect of the computational prediction of xenobiotic metabolism is the identification of the metabolically labile atom positions, also known as sites of metabolism (SoMs) (Kirchmair et al., 2012). Being able to predict SoMs is important because knowing an atom position in a molecule at which a metabolizing reaction is likely to occur usually provides a chemist with a good idea of the ensuing metabolite structure. Besides a range of commercial offerings, several freely available tools, such as SMARTCyp (Olsen et al., 2019), SOMP (Rudik et al., 2015), Xenosite (Zaretzki et al., 2013), and FAME 2 (Šícho et al., 2017), are able to predict SoMs with high accuracy (Tyzack and Kirchmair, 2018). FAME 2, which is used in the present work for SoM prediction, is a machine learning-based tool developed recently in our group. The extra trees classifier models of FAME 2, which are based on a set of 2D circular descriptors, were developed specifically to predict SoMs of metabolic reactions catalyzed by the CYP family of enzymes in humans. FAME 2 is highly accurate, achieving, on an independent test set, a Matthews correlation coefficient of 0.57 and an area under the receiver operating characteristic curve (AUC) of 0.91.

In contrast to *in silico* SoM prediction, computational prediction of the structures of metabolites lags behind with respect to prediction accuracy. In general, existing methods for predicting metabolite structures for xenobiotics are dominated by rule-based approaches. There are a number of well-established commercial tools for metabolite structure prediction, including Meteor Nexus (Lhasa Ltd.), a rule-based metabolite prediction software (Marchant et al., 2008). Meteor Nexus offers three different reasoning methods to prioritize the plethora of generated metabolites. The current default reasoning method is SoM scoring, which compares the SoM identified by the reaction rule to experimental data in order to assign scores to the predicted metabolites^1^. Other rule-based computational tools include TIMES (LMC; Mekenyan et al., 2004), which uses a heuristic algorithm to generate possible metabolic maps, and MetabolExpert (CompuDrug; Darvas, 1987).

In addition to commercial metabolite structure prediction tools, there is an increasing number of freely available options. Again, many of the available options rely primarily on a set of reaction rules to generate structures of possible metabolites. One well-known approach that has been around for some time is SyGMa (Ridder and Wagener, 2008), which in this work is used as a reference method. SyGMa predicts metabolites using knowledge-based reaction rules, some of which were derived from common knowledge of metabolism reactions and some of which were developed using the Metabolite Database (MDL Metabolite Database, Elsevier, 2001), for a total of 144 reaction rules covering both phase I and phase II metabolism. The predicted metabolites are ranked by empirical probability scores calculated based on the fraction of predicted metabolites produced by the particular reaction rule that match reported metabolites in the database. Using all 144 phase I and phase II reaction rules in up to three successive reaction steps, SyGMa was able to predict 68% of all known metabolites in the test set. In terms of ranking, SyGMa ranked 45% of the known metabolites in the test set in the top 10. The authors additionally examined SyGMa's potential usefulness for predicting CYP-mediated metabolism by evaluating its performance on a set of 127 single-step CYP-mediated reactions. Using only the 118 phase I reaction rules, which include but are not specific to CYP-mediated reactions, SyMGa was able to predict 84% of all known CYP-formed metabolites and predict 66% of the known metabolites within the top three ranked predicted metabolites. However, the proprietary nature of the dataset that was used to derive SyGMa's reaction rules and validate the method, not to mention the current unavailability of the dataset, hinders the reproducibility of the results as well as further use of the models derived from the data.

A recent, free software designed to predict metabolites from multiple sources and enzyme families is BioTransformer (Djoumbou-Feunang et al., 2019), which in this work is used as the second reference method. BioTransformer is a comprehensive metabolite prediction tool that contains a CYP metabolite prediction module (in addition to four other metabolite prediction modules). BioTransformer predicts CYP-formed metabolites using a knowledge-based approach combined with built-in CYP selectivity prediction by CypReact (Tian et al., 2018), a machine learning-based tool, as a precursor to metabolite prediction. Aside from the initial CYP isoform-specificity prediction, the basis of BioTransformer's CYP450 metabolite prediction module is a rule-based method whose reaction rules are derived partly from the metabolic reactions in MetXBioDB (Djoumbou-Feunang et al., 2019), a freely available database of metabolism reactions that was established in the context of developing BioTransformer. In the current version of BioTransformer, the predicted metabolites are not ranked. BioTransformer also offers an option for identifying metabolites based on masses from mass spectrometry data. On a test dataset of 60 parent molecules with a total of 180 known metabolites, BioTransformer's CYP450 metabolite prediction module achieved a recall of 0.90 and a precision of 0.46.

Another freely available metabolite prediction tool is MetaTox (Rudik et al., 2017), which encompasses both phase I and phase II metabolism and combines the prediction of the reaction class and the reacting atom in order to predict metabolites. Additionally, the open-source software Toxtree (Patlewicz et al., 2008) contains a metabolism prediction module called “SMARTCyp—Cytochrome P450-Mediated Drug Metabolism” that predicts SoMs using SMARTCyp (Rydberg et al., 2010) and then applies a small set of reaction rules to the predicted SoMs in order to predict metabolites.

Common to all modern approaches for metabolite prediction is that they remain challenged by the combinatorial explosion of predictions, in particular when looking at several generations of metabolites (Judson, 2014). It is not unusual for metabolite structure predictors to produce several pages full of predicted metabolites, a fact which is often and not without reason criticized, particularly by experts in metabolism. The key to tackling this problem lies in the development of approaches for the accurate ranking of metabolites according to their relevance in terms of metabolic rates and biological properties. A number of methods attempt to get a handle on the immense number of predicted metabolites by ranking their predictions according to various approaches.

Another option, which has primarily been implemented in commercial tools to date, is to use SoM prediction as a preliminary step to reduce the number of generated metabolites. Commercial tools for metabolite prediction that incorporate SoM prediction include ADMET Predictor (SimulationsPlus)^2^, which predicts SoMs and the corresponding metabolite structures for nine CYP isoforms, and StarDrop (Optibrium; Tyzack et al., 2016), whose “P450 metabolism” module predicts SoMs using quantum mechanical simulations and displays the structures of the metabolites corresponding to the predicted SoMs. In addition, META Ultra (MultiCASE Inc.; Klopman et al., 1994) predicts SoMs and metabolites, and MetaSite (Cruciani et al., 2005) was a SoM and CYP isoform selectivity prediction software that now also predicts metabolite structures^3^.

Few freely available metabolite prediction methods combine information on predicted SoMs with a rule set. MetaTox predicts reaction classes and reacting atoms (i.e., SoMs, in principle) separately for each parent molecule, then combines the predictions to generate metabolites. The probability that the metabolite is formed is calculated based on the predicted probabilities of the reaction class and of the SoM predicted with the SOMP method (Rudik et al., 2015). However, the validation of MetaTox considers the performance of the reaction class prediction and the reacting atom prediction separately, without evaluating the prediction of the metabolite structures themselves, and it is unclear how exactly the reaction class and reacting atom predictions are combined to generate a metabolite structure (Rudik et al., 2017). On the other hand, it is clear that SoM prediction is used directly as a prefilter before applying reaction rules in the SMARTCyp Toxtree module. However, a validation of this method has not been published.

In terms of the availability of rule sets for metabolite structure prediction, there are a few existing freely available collections of reaction rules described in an easily accessible, computer-readable format such as SMIRKS^4^, a reaction transform language within the Daylight system. One source of CYP reaction rules is the SMARTCyp Toxtree module, which uses 16 reaction rules and makes the SMIRKS freely available as part of the source code. A larger selection of reaction rules is provided in the freely available SyGMA Python package. The reaction rules are clearly separated into phase I and phase II rules; however, there is no indication of which of the 118 phase I reaction rules specifically describe CYP-mediated reactions. In addition, these rules were derived from a proprietary and no longer distributed dataset. BioTransformer offers a large number of CYP-specific biotransformation rules in SMIRKS format as well as additional constraint(s) for each rule as part of its Reaction Knowledgebase.

In this work, we present a multipronged approach to the prediction of metabolites formed by the CYP enzyme family in humans. In reference to FAME, we name this approach GLORY. One fundamental aspect of GLORY is a new, easily interpretable rule base for CYP metabolism that was developed solely from the scientific literature and basic chemistry knowledge, without relying on any dataset of metabolic reactions. In addition, we have examined the effect of using SoM prediction as a preliminary filter for the positions at which reaction rules are allowed to be applied and also as part of a new approach to ranking the predicted metabolites. GLORY therefore has two modes: MaxCoverage, which focuses solely on recall, and MaxEfficiency, which focuses more on precision. Further, we have validated GLORY on a new, high quality, manually curated dataset that is provided in the Supplementary Material.

## Results and Discussion

Two key aspects are at the core of GLORY, which aims to predict metabolites within the context of human, CYP-mediated metabolism: reaction rules and predicted SoMs. In terms of the rule-based aspect, GLORY uses reaction rules to convert parent molecules into their possible metabolites. To this end, we developed a collection of rules based entirely on the scientific literature to ensure that the rule set was not biased by any particular metabolism dataset. The information on the CYP-mediated reactions from the literature was combined with basic chemistry knowledge to develop SMIRKS to describe each reaction type. In some cases, such as for O-dearylation, multiple SMIRKS were required for a single reaction type, resulting in a total of 73 SMIRKS for the 61 reaction types present in our collection (Supplementary Table 1). We additionally use a simple binary distinction between common and uncommon reaction types, which were thoroughly discussed and distinguished from each other in Guengerich (2001), and which distinction we were able to extrapolate to the CYP-mediated reactions found elsewhere in the literature (see Methods for details). We do not use occurrence ratios calculated based on a given dataset in order to rank the predicted metabolites, due to the limited size, quality, and accessibility of existing datasets. Out of our collection of 61 CYP reaction types, 22 have been designated as common.

**Figure 1:**
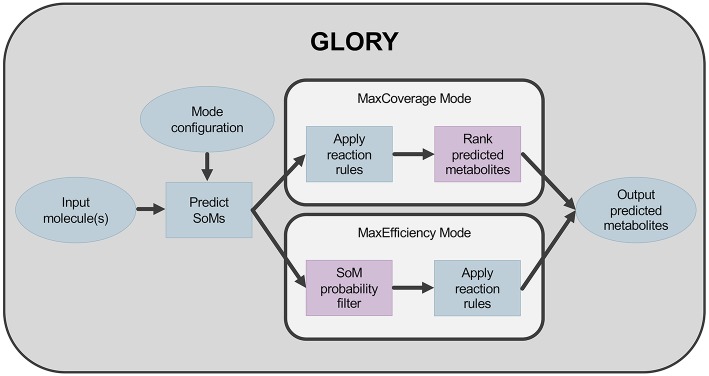
Workflow for GLORY indicating the difference between MaxCoverage mode and MaxEfficiency mode.

The second key aspect of GLORY is its use of the SoM probabilities predicted by FAME 2 for each heavy atom in a molecule to (i) reduce the false-positive prediction rate while maintaining an acceptable recovery rate and (ii) augment the ranking of predicted metabolites. In order to reduce the false-positive prediction rate, the possibility of utilizing a hard cutoff based on SoM probabilities was explored. This cutoff was used to determine at which atom positions the rules were allowed to be applied. In the context of GLORY, we have called this approach, in which SoM prediction is used as a preliminary filter, MaxEfficiency mode. In contrast, we designate the approach in which SoM probabilities are used for ranking metabolites derived for all positions in a molecule regardless of SoM probability the MaxCoverage mode. The difference in workflow between the two modes is illustrated in Figure 1.

### Datasets

To choose a SoM probability cutoff for the MaxEfficiency mode and develop a priority score to rank predicted metabolites, a large reference dataset was generated by combining the CYP metabolism data extracted from DrugBank (Wishart et al., 2018) and MetXBioDB. MetXBioDB is a recently published database of metabolic reactions, whose substrates are mainly comprised of xenobiotics and also include a few sterol lipids and mammalian primary metabolites, and whose reaction data came from the scientific literature as well as publicly available databases (Djoumbou-Feunang et al., 2019). In addition, a manually curated, high-quality dataset was compiled from the scientific literature for the validation of GLORY. This test dataset contains 29 parent molecules and a total of 81 metabolites, resulting in 2.79 metabolites per parent molecule on average. Importantly, any parent compounds that are in the test dataset were removed from the reference dataset before any analysis occurred. In total, the reference dataset contains 848 parent molecules and a total of 1,588 metabolites, for an average of 1.87 metabolites per parent molecule. Predictions could be made for 847 of 848 molecules in the reference dataset (one molecule could not be processed successfully with FAME 2; see Methods for details).

### MaxEfficiency Mode: Selection of a Cutoff for Metabolite Structure Generation Based on SoM Probability

In order to determine the effect of a SoM prediction-based prefilter on predicting preferably only the most relevant metabolites and reducing the number of false positive predictions, we tried several different cutoffs for the SoM probability that must be achieved by at least one atom involved in the reaction (as defined by the reaction's SMIRKS). For each heavy atom in a molecule, FAME 2 reports a probability between 0 and 1, corresponding to the fraction of trees of the extra trees classifier that predict that a particular atom is a SoM. The decision threshold in FAME 2 for whether or not an atom is considered likely enough to be a SoM to be designated as such was determined by the trained model to be 0.4 (Šícho et al., 2017).

We examined the effect of different SoM probability cutoffs using the reference dataset and selected the cutoff to be used in MaxEfficiency mode based on these results. In particular, we inspected the effect of the SoM probability cutoffs on precision and recall, which are defined as follows:

$$\begin{array}{l} Recall\;=\;TP\;/\;\left(\;TP\;+\;FN\right)\\ Precision\;=\;TP\;/\;\left(TP\;+\;FP\right) \end{array}$$

where TP is the number of true positive predictions, FP is the number of (putative) false positive predictions, and FN is the number of false negative predictions. In other words, recall measures the portion of known metabolites that were reproduced by the method and precision measures the fraction of all predicted metabolites that are represented in the dataset.

Here it is worth noting that the number of false positives, and the designation of a prediction as false positive, is especially dependent on the dataset that is being used for comparison. Many metabolites that are formed in humans have not yet been discovered, or their structures have not yet been exactly elucidated. Since even the highest-quality dataset is limited by the available experimental data, the reality is that the distinction between a real false positive prediction and the true positive prediction of an as yet unknown or unconformed metabolite may not be possible. Nevertheless, with this caveat, we evaluate our method based on the available data, including the putative false positives.

The purpose of the MaxEfficiency mode is to use the SoM probability cutoff to predict metabolites with increased precision compared to no cutoff (i.e., MaxCoverage mode). At the same time, however, we did not want to sacrifice too much in terms of recall, as it is still important to predict a molecule's actual metabolites even while reducing the number of putative false positive predictions.

For the purpose of metabolite prediction, we found that using FAME 2's decision threshold of 0.4 as the cutoff for SoM probability resulted in a relatively low recall of 0.65 (especially when compared to the recall of 0.83 achieved in MaxCoverage mode, as will be discussed later in this work). Hence, despite the increased precision afforded by a cutoff of 0.4, it was determined that this cutoff too greatly reduced the achieved recall. We therefore additionally tested lower SoM probability cutoffs (Table 1). Observing the trade-off between precision and recall with cutoffs ranging from 0.4 to 0.1 and comparing them to MaxCoverage mode, we determined that a SoM probability cutoff of 0.2, which resulted in a precision of 0.19 and a still-high recall of 0.75, offered the best compromise. A SoM probability cutoff of 0.2 for MaxEfficiency mode was therefore fixed based on the results shown in this section. Note that although all of the precision values shown in Table 1 are quite low, the precision of GLORY using a SoM probability cutoff is comparable to the precision of existing methods for metabolite structure prediction (see below for the results on the test dataset).

**Table 1:** Effect of different SoM probability cutoffs on precision and recall over the entire reference dataset.

**SoM Probability Cutoff^a^**	**0.4**	**0.3**	**0.2**	**0.1**	**None**
Precision	0.24	0.22	0.19	0.13	0.07
Recall	0.65	0.71	0.75	0.80	0.83

a *Note that 0.4 is the default decision threshold in FAME 2, a cutoff of none corresponds to MaxCoverage mode, and a cutoff of 0.2 was chosen for MaxEfficiency mode*.

### Development of a Priority Score to Rank Predicted Metabolites for MaxCoverage Mode

**Figure 2:**
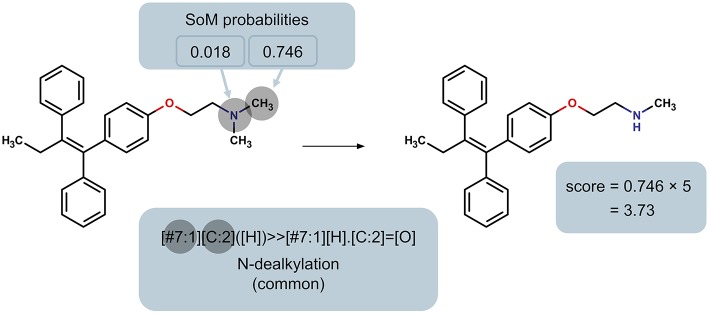
Illustration of the determination of the maximum SoM probability of all heavy atoms in the parent molecule that are matched by the reaction rule, using the N-dealkylation reaction rule (common reaction type; factor *F* = 5) as an example. This maximum probability is used to calculate the priority score of the product.

**Figure 3:**
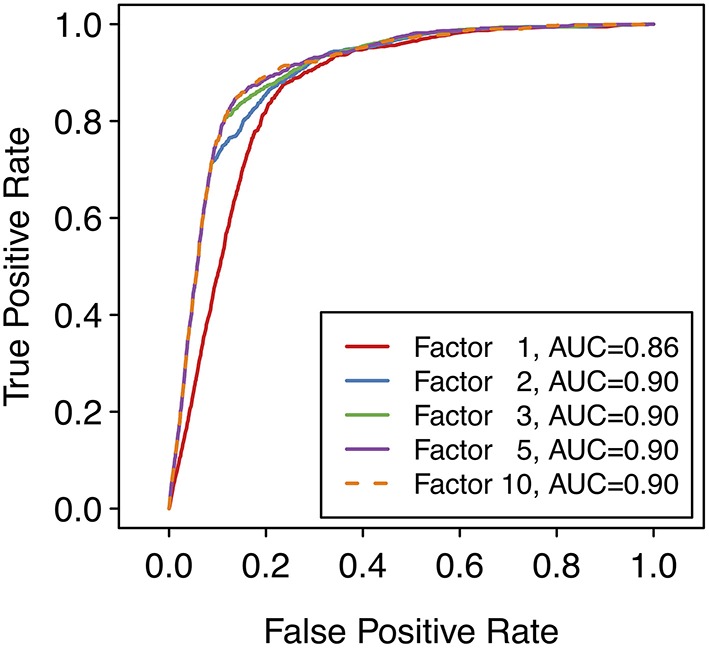
Receiver operating characteristic (ROC) curves over the entire reference dataset of 848 compounds with 1,588 known metabolites, with varying values of the factor used for common reaction types when calculating the priority score for each metabolite. Note that a factor of 1 means that only the SoM probability (i.e., the maximum SoM probability for all atoms that are matched by the SMIRKS) affects the priority score of the predicted metabolite, regardless of the reaction type. Note also that a ROC curve can be calculated despite there being no “true negative” predictions overall (all predicted metabolites are “positive” predictions). To generate the ROC curve, the false positive rate (FPR) is calculated at each score threshold. At each point, predictions with scores below the threshold are considered “negative” predictions and predictions with scores above the threshold are considered “positive” predictions. Hence the number of “true negative” predictions and therefore the FPR can be calculated for each point of the ROC curve.

In order to rank the predicted metabolites for a particular molecule, we developed a priority score for each predicted metabolite based on the SoM probability of the atoms involved in the transformation and whether the reaction type is common or not. Specifically, the SoM probability calculated by FAME 2 for all atoms in the parent molecule that are involved in a reaction as defined by the SMIRKS is considered, and the maximum SoM probability among these atoms is then incorporated into the score, as illustrated in Figure 2. The priority score was calculated using a simple formula:

$$\begin{array}{l} score_{\text{predictedmetabolite}}=\text{P}\times \text{F} \end{array}$$

where *P* is the maximum SoM probability out of the atoms in the parent molecule that were matched by the applied transformation and *F* is the factor according to whether the reaction type was designated as common or uncommon. In case the same predicted metabolite resulted from multiple transformations, the maximum priority score over all transformations leading to that prediction was used. A higher priority score is intended to indicate a higher likelihood of the prediction being true. For all uncommon reaction types, *F* = 1. The factor *F* for common reaction types affects the early enrichment of the predictions. Specifically, the early enrichment improves when common reaction types are given more weight in the score than uncommon reaction types, i.e. *F*_*common*_ > 1 (Figure 3). Based on an analysis of the receiver operating characteristic (ROC) curves and area under the ROC curves (AUC) for varying *F*_*common*_, shown in Figure 3, a factor of 5, resulting in an AUC of 0.90, was chosen. All subsequent results based on ranking the predicted metabolites therefore used *F*_*common*_ = 5 in the calculation of the priority score, and the priority score can therefore range from 0 to 5.

### Comparison of Performance on a New, Manually Curated Test Set

The performance of the MaxEfficiency and MaxCoverage modes of GLORY was evaluated on the curated test set of 29 parent molecules with a total of 81 metabolites. This evaluation includes a comparison with BioTransformer and SyGMa as well as an analysis of how well the scoring and ranking aspects of the different approaches work. Specifically, we employed the CYP450 module of BioTransformer and the phase I metabolism reactions of SyGMa (SyGMa does not feature a dedicated module for CYP metabolism, but phase I metabolism is carried out to a significant extent by CYP enzymes) for the comparison.

#### Analysis of MaxEfficiency Mode

GLORY's MaxEfficiency mode was designed to address the problem of low precision caused by a high number of putative false positive metabolite predictions. This general problem of an excess of predictions is well-documented for metabolite prediction tools (Judson, 2014). However, as mentioned above, it is important to note that the designation of predictions as false positive is particularly dataset-dependent.

As described previously, the MaxEfficiency mode uses a cutoff based on the SoM probabilities that FAME 2 predicts for each heavy atom in order to restrict the locations in the molecule at which the reaction rules are allowed to be applied. This SoM probability cutoff was set to 0.2 based on the analysis on the reference dataset; however, we also examine the effect of different SoM probability cutoffs using the high-quality test dataset in order to get a more complete picture of how much can be gained by a cutoff-based approach.

**Figure 4:**
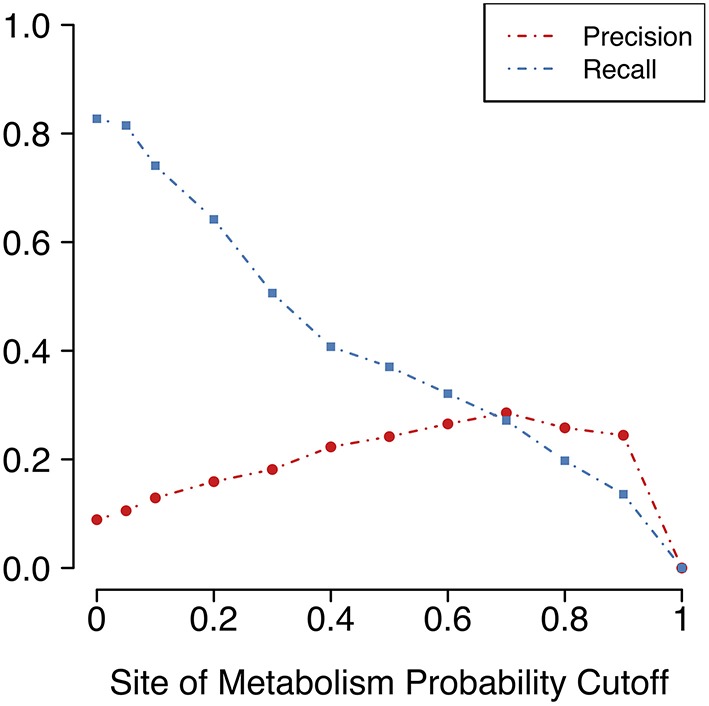
Precision (portion of predictions that are true positives) and recall (portion of known metabolites that are predicted) vary according to the cutoff for FAME 2's predicted SoM probability. A SoM probability cutoff of 0.4 corresponds to the decision threshold used in FAME 2. The SoM probability cutoff chosen for the MaxEfficiency mode of GLORY was 0.2.

As expected, using SoM predictions to confine the application of reaction rules to certain positions does involve a trade-off between precision and recall (Figure 4). Recall measures the portion of known metabolites that the method was able to reproduce, and precision measures the fraction of all predicted metabolites that are actually known metabolites (see previous section for definitions). The larger the SoM probability required to be present among the atoms involved in the transformation, the lower the recall but the higher the precision as measured across the entire test dataset. In addition, the larger the SoM probability cutoff, the more parent molecules there are for which no metabolite predictions can be made. Without any such cutoff and even up to a SoM probability cutoff of 0.2, metabolites can be predicted for all parent molecules in the test dataset. However, with a SoM probability cutoff of 0.3, no metabolites are predicted for two parent molecules, and this number increases to three for a cutoff of 0.4 (Supplementary Table 2). The number of molecules affected is small in this case, yet is approximately 10% of the size of the test dataset. Overall, as the cutoff increases, the total number of predicted metabolites decreases drastically (Supplementary Table 2).

**Figure 5:**
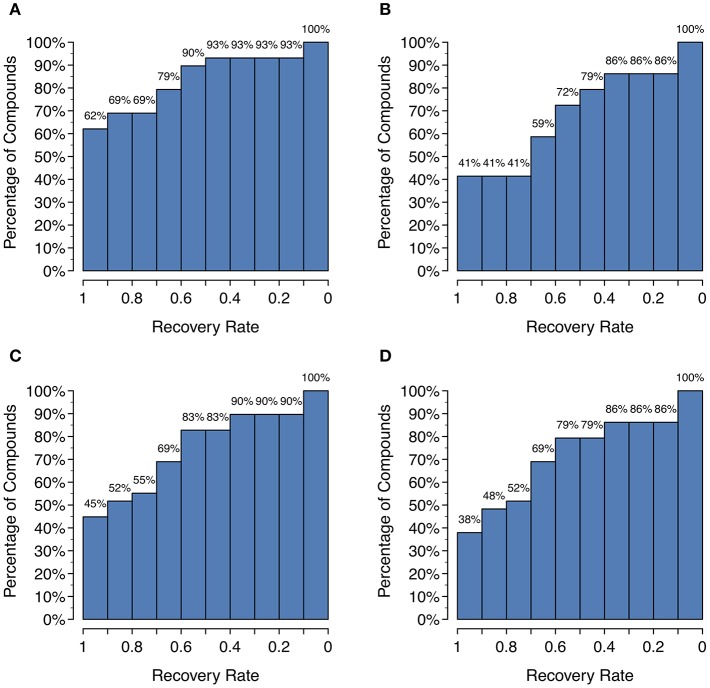
Histograms of the recovery rate of known metabolites broken down by parent compound: **(A)** GLORY in MaxCoverage mode, **(B)** GLORY in MaxEfficiency mode, **(C)** SyGMa, **(D)** BioTransformer. For example, a recovery rate of 0.5 indicates that for x% of all parent molecules, at least half of all recorded metabolites from the test dataset were predicted.

**Figure 6:**
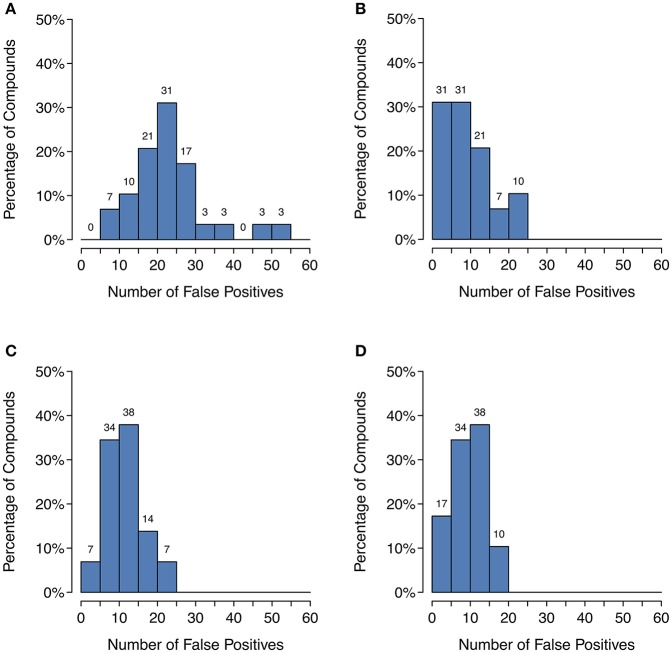
Histograms of the number of putative false positive predictions: **(A)** GLORY in MaxCoverage mode, **(B)** GLORY in MaxEfficiency mode, **(C)** SyGMa, **(D)** BioTransformer. These histograms use right-closed intervals.

Unfortunately, as Figure 4 shows, there is a large decrease in recall for a small increase in precision when using SoM probability cutoffs of 0.1 or greater. Looking more closely at the recovery rates per parent molecule, we see that GLORY's MaxEfficiency mode (using the selected cutoff of 0.2 as described above) can predict at least half of the known metabolites for 72% of the parent molecules in the test dataset, as opposed to 83% for SyGMa and 79% for BioTransformer (Figure 5). GLORY's MaxEfficiency mode can predict all known metabolites for 41% of the parent molecules in the test dataset, as opposed to 45% for SyGMa and 38% for BioTransformer. On the other hand, the number of putative false positives per parent molecule is brought to within the same range as was measured for SyGMa and BioTransformer (Figure 6). Using MaxEfficiency mode, most parent molecules have fewer than 10 putative false positives, which is also the case for BioTransformer but not quite the case for SyGMa (however, as mentioned above, SyGMa's rule base also includes rules for non-CYP-mediated phase I reactions).

Based on these results, it appears that using FAME 2's predicted SoM probabilities as a hard cutoff for metabolite prediction may not be sufficient for many use cases. However, the SoM predictions are useful for more than just as a hard cutoff, namely to rank the predicted metabolites, as will be shown in the next section.

#### Comparison of MaxCoverage Mode to SyGMa and BioTransformer

Neither SyGMa nor BioTransformer uses regioselectivity prediction as a prefilter before applying reaction rules. The same is true of MaxCoverage mode, which only uses SoM prediction in order to score and rank the predicted metabolites. Hence, we compared SyGMa and BioTransformer to GLORY's MaxCoverage mode in terms of recall, precision, and ability to rank the predicted metabolites.

A high recall is important for any use case of a metabolite structure predictor, but even more so for applications in which it is of utmost importance to not miss any physically existing metabolites, such as, for example, when attempting to identify metabolites based on MS data. GLORY's MaxCoverage mode performs well in terms of recall, with a recall of 0.83 compared to 0.74 and 0.72 for SyGMa and BioTransformer, respectively, across the entire test dataset (Table 2). A closer look at recall broken down to the level of the recovery rate of known metabolites for each parent molecule shows that GLORY is able to predict all known metabolites for 62% of the parent molecules, whereas SyGMa and BioTransformer achieve only 45% and 38%, respectively, in this regard (Figure 5). The number of parent molecules for which GLORY is able to predict at least half of the known metabolites is 90%, compared to 83% for SyGMa and 79% for BioTransformer (Figure 5).

**Table 2:** Evaluation results for SyGMa, BioTransformer, and GLORY's MaxCoverage and MaxEfficiency modes on the manually curated test dataset.

	**SyGMa**	**BioTransformer**	**GLORY, MaxCoverage mode**	**GLORY, MaxEfficiency mode^b^**
Precision	0.15	0.17	0.08	0.16
Recall	0.74	0.72	0.83	0.64
Total number of predicted metabolites	406	344	793	327
Number of successfully predicted reported metabolites^a^	60	58	67	52
Top-1	0%	N/A	68.97%	68.97%
Top-2	48.28%	N/A	72.41%	72.41%
Top-3	68.97%	N/A	75.86%	75.86%

a *The total number of reported metabolites in the dataset was 81*.

b *The SoM probability cutoff used for MaxEfficiency mode is 0.2, chosen based on the results of the analysis on the reference dataset. Data on the performance of MaxEfficiency mode with different SoM probability cutoffs are reported in Supplementary Table 2*.

Precision can be a useful metric for measuring how well a method is able to keep the number of putative false positive predictions under control. Precision was low across the board for metabolite prediction on the test dataset, with BioTransformer reaching the highest precision of the three tools at 0.17. SyGMa was close behind at 0.15, and GLORY's MaxCoverage mode lagged further behind at a precision of only 0.08 (Table 2). Again breaking this down to a slightly more detailed overview, we see that BioTransformer and SyGMa both always produce fewer than 25 putative false positives per parent molecule and, for the majority of parent molecules, fewer than 15 putative false positives or even, in the case of BioTransformer, fewer than 10 (Figure 6). GLORY in MaxCoverage mode, on the other hand, often produces so many predictions per parent molecule that there are up to 53 putative false positives per parent molecule in the test dataset and on average a relatively high number of putative false positive predictions compared to the other two tools (Figure 6).

In the case of the low precision observed for SyGMa, it is important to note that SyGMa's rule set is not specific to CYP-mediated metabolism but rather covers phase I metabolism in general. This could indicate that SyGMa might achieve higher precision if only the CYP-specific rules were used.

BioTransformer's CYP450 prediction module, which has the highest precision of all three methods, uses isoform prediction as a preliminary filter. Only the relevant reactions for the predicted metabolizing CYP isoform(s) are applied to the parent molecule, which could contribute to the observed precision.

Although the precision of MaxCoverage mode (as well as SyGMa and BioTransformer) was found to be low and high rates of false positive predictions are problematic in general, in the case of metabolite structure predictors a low precision is only problematic if there is no way to distinguish between the true positive and putative false positive predicted metabolites. This distinction can be achieved with a well-working ranking of the predicted metabolites, which circumvents the need to reduce the total number of predicted metabolites. Hence it is important that a metabolite prediction tool can rank the predicted metabolites in terms of likelihood of occurrence.

GLORY scores its predicted metabolites based partly on the maximum SoM probability of all the atoms involved in the reaction and also takes the type of reaction into account (see above for a more detailed description of the priority score). SyGMa uses empirical probability scores calculated based on the percentage of all predictions for each reaction rule that are found in the training dataset. SyGMa's scoring system thereby relies entirely on the discontinued Metabolite dataset. The scores generated by GLORY or by SyGMa can be used to rank the predicted metabolites for a given parent compound in terms of their likelihood of occurring. The current version of BioTransformer, on the other hand, does not score or rank its predictions.

We compared the ranking capability of GLORY's MaxCoverage mode with that of SyGMa. SyGMa was able to predict a known metabolite within the top three ranked positions for 69% of the parent molecules in the test dataset, whereas GLORY's MaxCoverage mode predicted a known metabolite within the top three predictions for 76% of the parent molecules (Table 2).

To look at the overall quality of the scoring as well as the ranking ability of SyGMa compared to GLORY, we generated ROC curves for each method using the score of each predicted metabolite as well as the rank of each predicted metabolite for a given molecule. The rank-based analysis corresponds better to the actual use case, in which it is desired to prioritize the predicted metabolites for a particular parent molecule, as opposed to over an entire dataset [note that SyGMa was originally only evaluated in terms of ranking per parent molecule (Ridder and Wagener, 2008)]. However, we additionally used the score-based ROC curve to visualize the performance of GLORY's priority score across the whole test dataset. To better allow for comparison of the ROC curves, false negatives were included in the ROC curves and thereby in the calculated AUCs by adding those molecules to the set of data points and artificially assigning them a score of 0 or rank of 1,000, as applicable, for the purpose of this evaluation.

**Figure 7:**
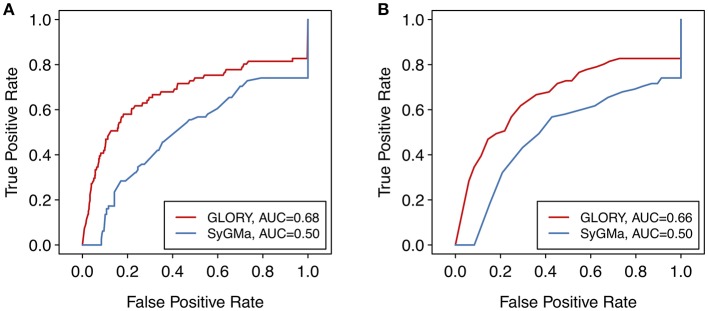
ROC curves over the entire test dataset comparing the **(A)** scoring and **(B)** ranking approaches of SyGMa to GLORY's MaxCoverage mode. For a better comparison of the two methods, false negatives were included in the ROC curve by assigning those data points a score of 0 or rank of 1,000, as applicable.

Though the AUC values are low, due in part to the inclusion of false negative data points in the ROC curves, the ROC curves show a much better earlier enrichment for GLORY than for SyGMa (Figure 7). SyGMa does not rank a known metabolite in the best-ranked position for any parent molecule in the test dataset (Table 2), which is reflected in the ROC curve. This decent early enrichment with GLORY, which is corroborated by the top-3 value, is a highly encouraging result indicating that the most likely predictions are closer to the top of the ranked list than the putative false positive predictions are.

One possible explanation for why SyGMa performs poorly in terms of scoring could be that its scoring scheme was derived from occurrence ratios in the Metabolite database and therefore optimized to predict the metabolites in that particular dataset. Although the Metabolite database was large, the authors of SyGMa report that the database was nevertheless biased toward compounds with one known metabolite and postulate that many of the metabolite profiles were incomplete (Ridder and Wagener, 2008). Our manually curated test dataset consists of parent molecules with metabolites that have been published since 2014, while SyGMa was developed using the 2001 version of Metabolite, so we assume that the overlap, if any, between SyGMa's training dataset and our test dataset is low. Without access to the dataset that was used to develop SyGMa's scoring methodology, it remains unclear how well the types of the reactions that lead to the metabolites in the test dataset were represented in their training dataset. Related to that, an additional downside of SyGMa's approach of basing their scoring approach on a database of metabolic reactions is that, since reaction rules can only be included if the database contains enough examples of a specific reaction type to calculate a probability score, more unusual reaction types or reaction types that are for some reason not well enough represented in the database may be missing from SyGMa's rule base (Ridder and Wagener, 2008).

There are several other differences in methodology between GLORY and SyGMa that could contribute to the difference in performance. Firstly, SyGMa does not specifically predict CYP-mediated metabolism but rather phase I metabolism in general, meaning that it could predict other phase I metabolites that are simply not present in the test dataset because they are not formed by CYPs. Second, in the current Python package implementation that was used for this validation, SyGMa does not appear to require its predicted metabolites to have a certain minimum size. Unlike GLORY, which does not output a potential metabolite if it has fewer than three heavy atoms, SyGMa predicts a handful of metabolites (across the whole test dataset) with only one or two heavy atoms.

### Computation Time

The run time for GLORY was measured on a workstation equipped with eight Intel(R) Core(TM) i7-4790 CPUs, 32 GB of main memory, and a Linux operating system. For the test dataset, the total run time (using eight cores) was 4.6 min in MaxCoverage mode and 4.3 min in MaxEfficiency mode (averaged over three runs). On average, the computation time per molecule required to predict metabolites was 10.9 s for MaxCoverage mode and 10.3 for MaxEfficiency mode (averaged over three runs).

## Methods

### Development of a Collection of Transformations

A collection of transformations, defined by SMIRKS and representing reaction types, was assembled based on known CYP-mediated reactions found in the literature (see Supplementary Material for details). The SMIRKS were defined to be as general as possible while being restricted to reasonable reaction chemistry, as indicated by the literature and common chemical knowledge. Therefore, if a reaction was found in the literature but it was not clear how the reaction would apply to other molecules besides the provided example, the reaction was excluded from the collection. This was the case for most reactions involving large ring systems as well as ring fusions and ring contractions. Specifically, the following types of reactions were excluded from our collection: reactions that appeared to be singleton reactions, reactions involving more than two fused rings that are not part of a steroid backbone, ring fusions, ring contractions, reactions in which the substrate or product is a radical, and reactions specifically indicated to have been found only in the case of plant CYP isozymes.

A few of the SMIRKS used to describe the transformations were taken from the Toxtree SMARTCyp module^5^. Most of the SMIRKS, however, were newly developed specifically for GLORY. When developing the SMIRKS expressions, care was taken to include as few atoms as possible in the explicit mapping, since SoM probabilities were considered for all atoms in the mapping.

Each reaction type was designated as either “common” or “uncommon.” Whenever possible, this label was assigned according to the reaction's classification by Guengerich in his 2001 review of CYP-mediated reactions (Guengerich, 2001), which explicitly divided the reactions into these two categories. If the reaction type was not described in that publication, a “common” or “uncommon” label was chosen based on extrapolation (on the basis of empirical similarity to reaction types present in the publication).

Our collection of CYP reaction rules consists of 61 reaction types. In some cases, multiple transformations were required to describe the same reaction type, leading to a total of 73 transformations in the collection of defined reactions. A full list of the reaction types and their SMIRKS can be found in Supplementary Table 1.

### Metabolite Prediction Program

Predicting the structures of the metabolites involves applying the reaction rules at all relevant positions. The relevant positions are determined by the reaction rule itself and, in the case of the MaxEfficiency mode, by the SoM probability predicted for each heavy atom. In MaxCoverage mode, the SoM probabilities are also used to score the predicted metabolites.

#### SoM Prediction With FAME 2

The SoM predictions were carried out using the FAME 2 software (Šícho et al., 2017), which included preprocessing of the molecules. The circCDK_ATF_6 trained model, which had the best average performance during the independent test set validation in Šícho et al. (2017), was used for the SoM prediction within GLORY.

#### Application of Transformations

The transformations of parent molecules into predicted metabolites based on the defined SMIRKS strings were performed using Ambit-SMIRKS [Kochev et al., 2018; Ambit-SMARTS Java Library, version 3.1.0. http://ambit.sourceforge.net/smirks.html (accessed Oct 4, 2017)]. Some transformations may result in multiple products. Products that contain fewer than three heavy atoms are not included in the set of predicted metabolites generated by GLORY.

When SoM prediction is used as a preliminary filter, a transformation rule is only applied at a particular location in the parent molecule if one of the heavy atoms involved is predicted to be a SoM with a probability over a certain threshold (see Results for more information on this threshold).

#### Scoring of Predicted Metabolites

The scoring of the predicted metabolites was based on SoM probability predictions and whether the reaction type was designated as common or uncommon. Each atom in the parent molecule was assigned a likelihood of being a SoM by FAME 2. When applying the transformations defined by SMIRKS, Ambit-SMIRKS maps the reactant portion of the defined transformation to any matching set of atoms in the parent molecule. Within this mapping, the maximum SoM probability was calculated and used to score the predicted metabolite that resulted from this particular transformation and mapping.

For each predicted metabolite, the priority score is calculated by multiplying the maximum SoM probability within the mapping with a factor *F* depending on whether the reaction type was classified as “common” or “uncommon.” Priority scores for the predicted metabolites therefore range from 0 to *F*_*common*_. The higher the score, the more likely the predicted metabolite is considered to be. See Results for further details on the selection of values for *F*.

If multiple transformations of a given parent molecule lead to the same metabolite structure, the priority score is calculated separately in each case and the highest score is retained. To calculate top-*k* values and rank-based ROC curves, it was necessary to rank the predicted metabolites for each parent molecule based on their priority scores. If different metabolites of the same parent compound have the same priority score, then they receive the same rank. In the case of a tie, one or more rank numbers, according to the number of tied predictions, following the tied rank are skipped. For example, if the highest score is 2.5 and two predicted metabolites both have this score, then both of these metabolites are assigned a rank of 1, no predicted metabolite is assigned a rank of 2, and the predicted metabolite(s) with the next highest score are assigned the rank of 3.

#### Program Output

The predicted metabolites are provided as an SD file with the following information for each predicted metabolite: rank (out of all predicted metabolites for a particular parent molecule), priority score, reaction name, and the InChI, SMILES, and ID of the parent molecule. If multiple transformations led to the same product, the highest priority score and the corresponding reaction name are reported. If the input consists of multiple molecules, the ID of a parent molecule is set to the molecule's position in the ordered list of input molecules (i.e., its position in the input file).

### Creation of the Reference Dataset

The reference dataset was made by combining the CYP metabolism data from DrugBank and MetXBioDB. The total size of the combined reference dataset, not including any metabolism information for any of the parent molecules contained in the manually curated test dataset, is 848 parent molecules and 1588 metabolites (an average of 1.87 metabolites per parent molecule).

#### DrugBank Dataset

The DrugBank database (DrugBank, version 5.1.2. https://www.drugbank.ca/ [accessed Jan 14, 2019]) was downloaded from the website. In addition to the database in XML format, the structures of all of the molecules, both parents and metabolites, were downloaded in SD format from the website (drug group “All” for the parent molecules).

Any parent or metabolite molecule without an available structure was ignored. One parent compound (DrugBank ID: DB09327) was ignored because its SMILES had two components of which the main component could not be unambiguously identified. All available generations of metabolism reactions were considered, as long as the reaction was annotated as mediated by one or more CYP isozymes. The enzymes for the reactions listed in DrugBank do not have any apparent species information, so all were assumed to be human and thereby relevant for this dataset.

For all CYP-mediated reactions, the reactant was considered to be the parent molecule and the product was considered to be a first-generation metabolite of that particular parent molecule. Any metabolite with the same InChI, ignoring stereochemistry information, as its parent molecule was removed from the set of metabolites for that parent molecule. Only those parent molecules with at least one valid metabolite were included in the final dataset.

Finally, the six parent molecules that are also present in the manually curated test dataset were removed from the DrugBank dataset prior to any evaluation, along with their corresponding metabolism information. These parent compounds were bupropion, ticlopidine, imipramine, ifosfamide, bosentan, and olanzapine.

After preprocessing, including removal of the overlap with the manually curated test dataset, the DrugBank dataset contained 364 parent molecules and 702 metabolites in total, with an average of 1.93 metabolites per parent molecule in the dataset.

#### MetXBioDB Dataset

The human, CYP-mediated reactions were extracted from the MetXBioDB dataset (MetXBioDB, version 1.0. https://bitbucket.org/djoumbou/biotransformerjar/src/master/database/ [accessed Jan 11, 2019]). As the only structural information provided in the MetXBioDB is in the form of InChIs and InChIKeys, any substrate or product without a reported InChI could not be considered. A lacking InChI was only the case for one out of 1468 CYP-mediated, human reactions in MetXBioDB.

Stereochemistry information was removed by generating InChIs without a stereochemistry layer, resulting in 751 CYP, human parent compounds in total. Of these, 259 are also present in the DrugBank dataset. For these overlapping parent compounds, 512 of 569 DrugBank metabolites are also in MetXBioDB, and MetXBioDB has an additional 93 metabolites for these overlapping parent compounds.

Eight parent compounds (olanzapine, bupropion, metoclopramide, bosentan, imipramine, ticlopidine, ifosfamide, and atomoxetine) from the manually curated test dataset were also present in the MetXBioDB dataset, only two of which (metoclopramide and atomoxetine) were not also present in the DrugBank dataset. These parent compounds and the corresponding metabolism data were removed from the MetXBioDB dataset.

After preprocessing, including removal of the overlap with the manually curated test dataset, the MetXBioDB dataset contained 743 parent molecules and 1385 metabolites in total, with an average of 1.86 metabolites per parent molecule in the dataset.

#### Merger of the DrugBank and MetXBioDB Datasets

The DrugBank dataset and the MetXBioDB dataset were combined to form the reference dataset via a straightforward consolidation of the parent and metabolite information. All molecule comparisons occurred using InChIs generated without stereochemistry information. For any parent molecule that was present in both the DrugBank and the MetXBioDB datasets, which was the case for 259 parent molecules, the sets of metabolites from both datasets were combined, disregarding stereochemistry, to yield the final set of metabolites for that parent molecule in the reference dataset.

### Creation of the Manually Curated Test Dataset

A new dataset for testing GLORY was manually assembled from the scientific literature. The data were extracted from publications on metabolism that were found in two journals: *Xenobiotica* and *Drug Metabolism and Disposition*. The time frame considered was from January 2014 to June 2018 for *Xenobiotica* and from January 2014 to June 2017 for *Drug Metabolism and Disposition*.

Publications were chosen and the metabolism information they contain included in the dataset if the following criteria were fulfilled:

1. The publication must contain a figure that depicts the metabolism scheme and includes the chemical structures of the parent compound and the first-generation metabolites.
2. The metabolism data must have been experimentally determined from a human source (i.e., either humans, human cells, or recombinant human CYP enzymes). If some but not all of the data were from humans, any non-human metabolites in the metabolism scheme were excluded from the dataset.
3. For at least 75% of all of the first-generation human metabolites depicted in the metabolism scheme (note that any metabolite that is depicted as merely being an intermediate is not considered), the following two criteria must be satisfied. First, the identity of the enzyme(s) responsible for the formation of the metabolite must be known. For this purpose, it is sufficient to know whether or not this metabolite is formed by CYPs. Second, the exact chemical structure, including the connectivity of all atoms, of the metabolite must be known. There is one exception to this rule: If the metabolite is known to not be CYP-formed, then the exact structure is not relevant and the metabolite is counted anyway.

Based on these criteria, 29 metabolism schemes containing at least one human, CYP-formed first-generation metabolite with a fully defined structure were found and included in the dataset. For these 29 parent molecules, there are 81 metabolites in total that fulfill the criteria (first-generation, human, CYP, fully defined structure) for inclusion in the dataset. Note that only first-generation metabolites are included in the dataset. Note also that intermediates, as depicted in the metabolism scheme, are not included in the dataset. Instead, the first non-intermediate metabolite in the pathway is used.

The SMILES for the metabolites were generated using ChemSpider (ChemSpider. http://www.chemspider.com/ [accessed Feb 13, 2019]). Consistency of stereochemistry information between parents and their metabolites was maintained.

### Validation of Metabolite Structure Predictors

Predicted metabolites were compared to known metabolites from the reference and test datasets using their InChIs. The InChIs used for this comparison were generated without stereochemistry information using CDK (Willighagen et al., 2017; Chemistry Development Kit, version 2.0. https://cdk.github.io/ [accessed Nov 3, 2017]).

During the validation, a predicted aldehyde metabolite was considered equivalent to the corresponding carboxylic acid, because there is evidence that some percentage of an aldehyde metabolite acts as an intermediate that is further oxidized to a carboxylic acid without leaving the CYP enzyme active site (Bell-Parikh and Guengerich, 1999).

In the case of one parent molecule in the reference dataset, no predictions could be made because the parent molecule contains boron. FAME 2 is unable to make predictions for molecules containing boron because no boron-containing molecules were present in the dataset used to train the model.

The SyGMa predictions were carried out in Python using the SyGMa Python package (SyGMa, version 1.1.0), and RDKit (RDKit: Open-Source Cheminformatics, version 2017_03_01, 2017). Only the phase I reaction rule set was used and one reaction cycle was applied.

The BioTransformer predictions were performed using the CYP450 mode of the BioTransformer (BioTransformer, version 1.0.8. https://bitbucket.org/djoumbou/biotransformerjar/src/master/ [accessed Feb 5, 2019]) command line tool. BioTransformer was run individually for each parent compound using single SMILES input.

The ROC curves were generated using the ROCR R package (Sing et al., 2005; ROCR, version 1.0-7, 2015). When false negative data points were added to the curve, these data points were assigned a score of 0 or a rank of 1,000, respectively, depending on whether the ROC curve represented scores or ranks.

## Conclusions

We have developed GLORY, a new tool for predicting the structures of human metabolites formed by CYPs. GLORY incorporates two key ideas: a literature-based collection of CYP-mediated reaction rules and SoM prediction, which was used particularly auspiciously to develop a new scoring approach for the predicted metabolites.

For GLORY, we developed a new collection of 73 reaction rules, describing 61 reaction types, for CYP-mediated metabolism. In developing this collection, we prioritized the reproducibility of our rule set and therefore based the rules on the scientific literature rather than on any dataset. In addition to the rules themselves, each reaction type was designated as either common or uncommon, again based on the scientific literature rather than on any dataset.

In addition, we have devised a priority score for predicted metabolites based on predicted SoM probabilities and the simple, literature-based distinction between common and uncommon reaction types. Hence neither our rule set nor our scoring approach is directly based on any dataset of metabolic reactions, setting our approach apart from other tools, for example SyGMa, which uses reaction rules and occurrence ratios derived from a proprietary dataset, and BioTransformer, whose rules were to some extent based on a freely available dataset.

GLORY has two modes: MaxEfficiency, which uses SoM prediction as a prefilter for the positions in a molecule at which reactions are allowed to occur, and MaxCoverage, which does not use a prefilter and instead focuses on high recall and an accurate ranking of the predicted metabolites. Using SoM prediction as a preliminary filter, i.e., in MaxEfficiency mode, does not work as well as might be expected in terms of reducing the number of putative false positive predictions while still keeping a high rate of recovery of reported metabolites. However, by developing a priority score for the predicted metabolites using SoM prediction combined with a simple binary distinction between common and uncommon reaction types, we are able to rank the metabolites predicted by MaxCoverage mode to the extent that GLORY can predict at least one known metabolite within the top three ranked positions for 76% of the molecules in the independent test set while achieving a recall of 0.83. GLORY's MaxCoverage mode outperforms both SyGMa and BioTransformer in terms of recall and outperforms SyGMa in terms of ranking (BioTransformer does not currently rank its metabolite predictions). One use case for the MaxCoverage mode could be, for example, identifying metabolites from mass spectrometry data.

Along with the collection of reaction rules, we provide a new, manually curated test dataset for free use as a benchmark dataset. In addition, GLORY is freely available as a web server at https://acm.zbh.uni-hamburg.de/glory/.

Importantly, the concept of GLORY is such that it can be extended to predict metabolites formed by enzymes not belonging to the CYP family. The enzymes that this approach can be expanded to is limited, in principle, only by the extent of the available data and the coverage of the relevant metabolic reactions by SoM prediction tools.

## Data Availability

Publicly available datasets were analyzed in this study. This data can be found here: https://bitbucket.org/djoumbou/biotransformerjar/ and https://www.drugbank.ca/.

## Author's Note

The GLORY web service is available at the following address: https://acm.zbh.uni-hamburg.de/glory/.

## Author Contributions

CdBK and JK: conceptualization; CdBK, CS, and JK: methodology; CdBK, CS, MŠ, NK, and NJ: software development; CdBK: validation; JK and DS: resources; CdBK: data curation; all authors: writing—original draft preparation; CdBK: visualization; DS, NJ, and JK: supervision; JK: project administration; DS and JK: funding acquisition.

### Conflict of Interest Statement

NJ is a founder and co-owner of Ideaconsult Ltd. and has been the technical manager of the company since 2009. NK works for Ideaconsult Ltd. on a part-time basis. The remaining authors declare that the research was conducted in the absence of any commercial or financial relationships that could be construed as a potential conflict of interest.

## Footnotes

^1^Meteor Reasoning Methodologies, Lhasa Limited, https://www.lhasalimited.org/products/meteor-reasoning-methodologies.htm

^2^ADMET Predictor Metabolism Module, SimulationsPlus, https://www.simulations-plus.com/software/admetpredictor/metabolism/

^3^MetaSite, https://www.moldiscovery.com/software/metasite/

^4^SMIRKS—A Reaction Transform Language, Daylight, http://www.daylight.com/dayhtml/doc/theory/theory.smirks.html

^5^Toxtree Module: SMARTCyp—Cytochrome P450-Mediated Drug Metabolism, http://toxtree.sourceforge.net/smartcyp.html

## Supplementary Material

Supplementary Tables Reaction rules and additional evaluation results (DOCX).

Supplementary Data Sheet 1 Test dataset containing SMILES for parent molecules and metabolites as well as publication references (CSV).

Supplementary Data Sheet 2 Reference dataset containing SMILES (with stereochemistry information), InChI (without stereochemistry information), and DrugBank and MetXBioDB identifiers for parent molecules and metabolites (JSON).

## Abbreviations

AUC: area under the receiver operating characteristic curve
CYP: cytochrome P450
ROC: receiver operating characteristic
SoM: site of metabolism.
