# Supplementary material for: GLORY: Generator of the Structures of Likely Cytochrome P450 Metabolites Based on Predicted Sites of Metabolism
Source: Front Chem. 2019 Jun 12;7:402. doi: 10.3389/fchem.2019.00402 (PMC6582643; doi:10.3389/fchem.2019.00402)
Supplement: Supplementary Tables — Reaction rules and additional evaluation results (DOCX). [file Table_1.DOCX]

***Supplementary Material***

**Supplementary Table 1: Reaction Rules for GLORY, Including the SMIRKS, Sources, and Common vs Uncommon Label.**

| **Reaction Name** | **Source^a^** | **Common/ Uncommon** | **SMIRKS** |
| --- | --- | --- | --- |
| aliphatic hydroxylation | (1) | common | "[C;X4:1][H:2]>>[C:1][O][H:2]" |
| aliphatic hydroxylation with allylic rearrangement | (2) | uncommon^‡^ | "[C;!$(C(=C)CC=C);X3:1]=[C;X3:2][C;!$(C(C=C)C=C);X4:3]([H])>>[C:1](O)-[C:2]=[C:3]" |
|  |  |  | "[C;X3:1]=[C;X3:2][C;$(C(C=C)C=C)X4:3]([H])([H])>>[C:1](O)-[C:2]=[C:3]" |
| aromatic hydroxylation | (1) | common | "[c:1][H:2]>>[c:1][O][H:2]" |
| aromatic hydroxylation with NIH shift | (1, 3) | common | “[c:1]([H:5])[c;$(c1c([H])c([H])[c;H0]c([H])c([H])1):2][CH3,Br,Cl:3]>>[c:1]([*:3])[c:2][O][H:5]” |
| aromatic hydroxylation of pyrazolone | other^b^ | common^‡^ | "[#6;$([#6]1[#6](=O)[#7][#7][#6]:,=1),$([#6]1:,=[#6][#6](=O)[#7][#7]1):1][H:2]>>[#6:1][O][H:2]" |
| hydroxylation of cyclopropane | (2) | uncommon^‡^ | "[C:1]1[C:2][C:3]1[C:4]([H])>>[C:1](O)[C:2][C:3]=[C:4]" |
| amine hydroxylation | (1) | common | "[N:1]([H:3])[#6:2]>>[N:1]([O][H:3])[#6:2]" |
| N-dealkylation | (1) | common | "[#7:1][C:2]([H])>>[#7:1][H].[C:2]=[O]" |
| N-dealkylation of piperazine | (4) | common^‡^ | “[*;!#1:1][N;X3:2]1[C:3][C:4][N;X3:5][CH2][CH2]1>>[*:1][N:2][C:3][C:4][N:5]” |
| N-dealkylation of morpholine | (4) | common^‡^ | [N;X3;$(N1CCOCC1):1][CH2;$(C1NCCOC1)][CH2;$(C1OCCNC1)][O;$(O1CCNCC1):4]>>[N:1].[O:4] |
| S-dealkylation | (1) | common | “[#16:1][C:2]([H])>>[#16:1]([H]).[C:2]=[O]” |
| O-dealkylation of methylenedioxyphenyl | (5) | common^‡^ | “[O$(O1c2ccccc2OC1):1][C:2]([H])([H])[O$(O1c2ccccc2OC1):3]>>[O:1]([H]).[C:2](=O)[O-].[O:3]([H])” |
| S-oxidation | (1) | common | "[#16:1]>>[#16:1](=[O])" |
| N-oxidation | (1) | common | "[#7;X3,X2;H0:1][#6:2]>>[#7+:1]([O-])[#6:2]" |
| P-oxidation | (1) | common | “[#15;X3:1]>>[#15;X4:1]=[O]” |
| aldehyde oxidation to carboxylic acid | (1) | common | "[C:1]([H])=[O:2]>>[C:1](O)=[O:2]" |
| aldehyde oxidation to olefin plus HCO2H | (1) | uncommon | "[C:1]([H])[C:2]([H])[C:3]([H])=[O:4]>>[C:1]=[C:2].[C:3](O)=[O:4]" |
| olefin oxidation | (1) | common | “[C:1]([C:3])([C:4])=[C:2]([C:5])([C:6])>>[C:1](=O)([C:3])[C:2]([C:4])([C:5])([C:6])” |
| acetylene oxidation | (1) | common | “[#6:3][C:1]#[C:2][#6:4]>>[#6:3][C:1]([#6:4])=[C:2](=O)” |
|  |  |  | “[#6:3][C:1]#[C:2][#6:4]>>[#6:3][C:1]([#6:4])[C:2](=O)[O]” |
| oxidation of indole | (1) | uncommon | "[c;$(c1cc2ccccc2n1),$(c1c2ccccc2nc1):1]([H])=,:[c:2]-,:[n:3]>>[C:1](=[O])-[C:2]-[N:3]" |
| alcohol oxidation | (1) | common | "[C:1]([H])[O:2][H]>>[C:1]=[O:2]" |
| oxidation of 4-substituted phenol to quinone | (6) | uncommon^‡^ | “[c:1]1([O:7][H])[c:2][c:3][c;X3:4]([!C:8])[c:5][c:6]1>>[C:1]1(=[O:7])-[C:2]=[C:3]-[C;X3:4](=O)-[C:5]=[C:6]-1.[!C:8]” |
| oxidation of 4-substituted anisole to quinone | (6) | uncommon^‡^ | “[c:1]1([O:7][C:9])[c:2][c:3][c;X3:4]([!C:8])[c:5][c:6]1>>[C:1]1(=[O:7])-[C:2]=[C:3]-[C;X3:4](=O)-[C:5]=[C:6]-1.[!C:8].[C:9]” |
| oxidation of 4-substituted phenol to quinone if the substituent is not a leaving group | (6) | uncommon^‡^ | “[c:1]1([O:7][H])[c:2][c:3][c;X3:4]([C:8])[c:5][c:6]1>>[C:1]1(=[O:7])-[C:2]=[C:3]-[C:4]([C:8])(O)-[C:5]=[C:6]-1” |
| oxidation of 4-substituted phenol to quinone imine  (dehydrogenation) | (7) | uncommon^‡^ | "[c:1]1([O:7][H])[c:2][c:3][c;X3:4]([N:8][H])[c:5][c:6]1>>[C:1]1(=[O:7])-[C:2]=[C:3]-[C;X3:4](=[N:8])-[C:5]=[C:6]-1" |
| oxidation of 4-substituted anisole to quinone if the substituent is not a leaving group | (6) | uncommon^‡^ | “[c:1]1([O:7][C:9])[c:2][c:3][c;X3:4]([C:8])[c:5][c:6]1>>[C:1]1(=[O:7])-[C:2]=[C:3]-[C:4]([C:8])(O)-[C:5]=[C:6]-1.[C:9]” |
| oxidation of 1,4-dihydropyrridines | (1) | common | "[N;X3:1]1([H])[#6:2]=[#6:3][#6;X4:4]([H])[#6:5]=[#6:6]1>>[n;H0:1]1=[#6:2][#6:3]=[#6:4][#6:5]=[#6:6]1" |
| aliphatic epoxidation | (1) | common | "[C:1]=[C:2]>>[C:1]1[C:2][O]1" |
| N-dearylation | (1) | uncommon | “[c;R1:1]1[c;R1:2][c;R1:3][c;R1:4][c;R1:5][c;R1:6]1[N:7][c:8]>>[C:1]1=[C:2]-[C:3]-[C:4]=[C:5]-[C:6]1=[O].[c:8][NH2:7]” |
| O-dearylation | (1) | uncommon | “[c;R1:1]1[c;R1:2][c;R1:3][c;R1:4][c;R1:5][c;R1:6]1[O:7][c:8]>>[C:1]1=[C:2]-[C:3]-[C:4]=[C:5]-[C:6]1=[O].[c:8][O:7]” |
|  |  |  | “[c;R1:1]1[c;R1:2][c;R1:3][c;R1:4][c;R1:5][c;R1:6]1[O:7][c;R1:8]2[c;R1:9][c;R1:10][c;R1:11][c;R1:12][c;R1:13]2>>[C:1]1=[C:2]-[C:3]-[C:4]=[C:5]-[C:6]1=[O:7].[C:8]2(=[O])-[C:9]=[C:10]-[C:11]-[C:12]=[C:13]-2” |
|  |  |  | “[c;R1:1]1[c;R1:2][c;R1:3]([O:20][H])[c;R1:4][c;R1:5][c;R1:6]1[O:7][c:8]>>[C:1]1=[C:2]-[C:3](=[O:20])-[C:4]=[C:5]-[C:6]1=[O].[c:8][O:7]” |
|  |  |  | “[c;R1:1]1[c;R1:2][c;R1:3]([O:20][H])[c;R1:4][c;R1:5][c;R1:6]1[O:7][c;R1:8]2[c;R1:9][c;R1:10][c;R1:11][c;R1:12][c;R1:13]2>>[C:1]1=[C:2]-[C:3](=[O:20])-[C:4]=[C:5]-[C:6]1=[O:7].[C:8]2(=[O])-[C:9]=[C:10]-[C:11]-[C:12]=[C:13]-2” |
| deformylation | (1) | uncommon | "[C:1]([H])-[C:2]([H])-[C:3]=[O:4]>>[C:1]=[C:2].[C:3]=[O:4]" |
| oxidative desulfuration of phosphor | (8) | common^‡^ | "[*:1][P:2](=S)([*:3])[*:4]>>[*:1][P:2](=O)([*:3])[*:4]" |
| desulfuration of carbon | (8) | uncommon^‡^ | “[*:1][C:2](=S)[*:3]>>[*:1][C:2](=O)[*:3]” |
| reduction of N-oxide | (1) | uncommon | “[#7+;X4:1]([O-])>>[#7;X3:1]” |
| reduction of RNOR | (1) | uncommon | “[#8;$([#8][#6]):1][#7:2]:,=[#6:3]>>[#8:1]([H]).[#7:2]([H])([H])-[#6:3]([H])” |
|  |  |  | “[#8;$([#8][#6]):1][#7:2][#6;!X4:3]>>[#8:1]([H]).[#7:2].[#6:3]=[O]” |
|  |  |  | “[#8;$([#8][#6]):1][#7;$([#7][#6]):2]>>[#8:1]([H]).[#7:2]([H])” |
| reduction of nitro group | (1) | uncommon | "[N;X3:1](=O)=[O]>>[NH2:1]" |
|  |  |  | "[N+;X3:1](=O)[O-]>>[NH2:1]" |
| reduction of C- or N-nitroso compound | (1) | uncommon | “[C,N:1][N;X2:2](=O)>>[C,N:1][N;H2:2]” |
| azo reduction | (1) | uncommon | “[#6:1][N:2]=[N:3][#6:4]>>[#6:1][NH2:2].[NH2:3][#6:4]” |
| hydrazine reduction | (1) | uncommon | “[NX3:1]-[NX3:2]>>[N:1]([H]).[N:2]([H])” |
| alkyl oxidative dehalogenation | (1) | common | “[C:1]([H])[F,Cl,Br:2]>>[C:1]=[O].[F,Cl,Br:2]” |
| benzyl oxidative dehalogenation | (1, 9) | uncommon | “[c;$([c;!H]1ccccc1),$(c1[c;!H]cccc1),$(c1c[c;!H]ccc1),$(c1cc[c;!H]cc1),$(c1ccc[c;!H]c1),$(c1cccc[c;!H]1):1][F,Cl,Br,I:2]>>[c:1][O].[F,Cl,Br,I:2]” |
| reductive dehalogenation | (1) | uncommon | “[C:1]([F,Cl,Br:3])[C:2]([F,Cl,Br:4])>>[C:1]=[C:2].[*:3].[*:4]” |
| alkyl dehydrogenation | (1) | uncommon | “[C:1]([H])-[C:2]([H])>>[C:1]=[C:2]” |
| dehydrogenation of N-C bond | (8) | uncommon^‡^ | “[N;X3:1]([H])[C;!H3:2][H]>>[N:1]=[C:2]” |
|  |  |  | "[#7;X3:1]([H]):,-[#6;!H3:2]([H]):,=[#6:3]-[C:4]([H])>>[#7:1]=[#6:2]-[#6:3]=[C:4]" |
|  |  |  | “[N;X3:1][C:2][H]>>[N+:1]=[C:2]” |
| oxidative ether cleavage  (O-dealkylation) | (1) | common | "[#6:1][O:2][C:3]([H])>>[#6:1][O:2].[C;X3:3](=O)" |
| oxidative ester cleavage | (1) | uncommon | "[C$(C(O)([#6])=O):2][O:3][C:4][H]>>[C:2][O:3].[C:4]=[O]" |
| monothiophosphate ester cleavage | (8) | uncommon^‡^ | “[S:1]=[P$(P(O)(O)=S):2][O:3][#6:4]>>[S:1]=[P:2][O:3].[#6:4][O]” |
|  |  |  | “[S:1]=[P$(P(O)(O)=S):2][O:3][#6:4]>>[S:1].[O]=[P:2][O:3].[#6:4][O]” |
| phosphoester cleavage | (1) | uncommon | “[O:1]=[P$(P(O)(O)=O):2][O:3][#6:4]>>[O:1]=[P:2][O:3].[#6:4][O]” |
| carbamate cleavage | (8) | uncommon^‡^ | “[#7:1][C;$([C](O)=O):2][O:3][C:4]>>[#7:1][H].[C:2]=[O:3].[C:4][O]” |
| carbamide cleavage | (2) | uncommon^‡^ | “[N:1][C;$([C](N)(N)=O):2][N:3]>>[N:1][C:2].[N:3]” |
| oxidation of N-nitrosamine | (1) | uncommon | “[N$(N(C)C):1]([C:3][H])[N$(N(N)=O):2]>>[N:1]([H])([H]).[N:2]([O-]).[C:3](=O)” |
| scission of unsaturated fatty acid peroxides | (1, 2) | uncommon | "[C:1]([H])=[C:2]-[C:3]=[C:4]-[C:5]-[O:6]([O])>>[C:1]=[C:2][C:3](O)[C:4]1-[C:5]-[O:6]1" |
|  |  |  | "[C:1]([H])=[C:2]-[C:3]=[C:4]-[C:5]-[O:6]([O])>>[C:1]([O])[C:2]=[C:3][C:4]1-[C:5]-[O:6]1" |
| dehydration of an aldoxime to a nitrile | (1) | uncommon | “[C:1]([H])=[N:2][O]([H])>>[C:1]#[N:2]” |
| cyclization to 6-membered lactone | (10) | uncommon | “[C:1]([H])([OH])[#6:2][#6:3][#6:4][C;$(C=O):5][O:6][*:7]>>[C:1]1[#6:2][#6:3][#6:4][C;$(C=O):5][O:6]1.[*:7]” |
| cyclization to 5-membered lactone | (1) | uncommon | “[C:1]([H])[c:2][c:3][C;$(C=O):4][O:5][C,#1:6]>>[C:1]1[c:2][c:3][C:4][O:5]1.[*:6]” |
| cyclization to 6-membered NCN ring | (2) | uncommon | “[N;X3:1]([H])~[*:2]~[*:3]~[*:4]~[N:5]-[C:6]([H])>>[N:1]1~[*:2]~[*:3]~[*:4]~[N:5]-[C:6]1” |
| cyclization to 5-membered NCN ring | (2) | uncommon | “[N;X3:1]([H])~[*:2]~[*:3]~[N:5]-[C:6]([H])>>[N:1]1~[*:2]~[*:3]~[N:5]-[C:6]1” |
| cyclization to furan | (1) | uncommon | “[O:1]=[C;R1:2][C;R1:3]=[C:4][C:5]([H])[H]>>[O:1]1[C:2]=[C:3][C:4]=[C:5]1” |
| cyclobutamine expansion | (1) | uncommon | "[C:1]1-[C:2]-[C:3]-[C$(C1(C)CCC1):4]1[N:5]([H])>>[C:1]1-[C:2]-[C:3]-[C:4]=[N+:5]1" |
| oxidation of spiro[2,5]oxane | (11) | uncommon | "[C:1]1[C:2]2([C:3][C:4]2)[C:5]([H])[C:6][C:7][C:8]1>>[C:1]1[C:2]2(O)[C:3][C:4][C:5]2[C:6][C:7][C:8]1" |
| D-homoannulation of 17 alpha-ethinyl steroids | (1) | uncommon | "[C$([#6R1]~1~[#6R1]~[#6R1]~[#6R2]~2~[#6R2]~1~[#6R1]~[#6R1]~[#6R2]~3~[#6R2]~2~[#6R1]~[#6R1]~[#6R2]~4~[#6R1]~[#6R1]~[#6R1]~[#6R1]~[#6R2]~3~4):1]1([O:6][H])([C:7]#[C:8])[C:2][C:3][C:4][C:5]1>>[C:1]1(=[O:6])[C:7](=[C:8](O))[C:2][C:3][C:4][C:5]1" |

^a^ Note that the source(s) provided for each reaction type is not an exhaustive list. Many reaction types were found in multiple publications. When listing the source in this table, priority was given to the 2001 review by FP Guengerich (1) (because that is where the common/uncommon designation came from) and a second source only provided if it provided additional information used in the development of the SMIRKS. If the reaction was not found in reference 1, then the most general source that was applicable to the development of the SMIRKS was provided.

^b^ Special case of aromatic hydroxylation. This additional reaction type was needed because pyrazolone is not recognized as aromatic by CDK and Ambit SMIRKS.

^‡^ This designation was based on extrapolation, as this reaction type was not included in the 2001 review by FP Guengerich (1).

**Supplementary Table 2: Evaluation Results for GLORY in MaxEfficiency Mode with Varying Site of Metabolism (SoM) Probability Cutoffs on the Manually Curated Test Dataset.**

| **SoM Probability Cutoff** | **0.4** | **0.3** | **0.2** | **0.1** |
| --- | --- | --- | --- | --- |
| Precision | 0.22 | 0.18 | 0.16 | 0.13 |
| Recall | 0.41 | 0.51 | 0.64 | 0.74 |
| Total number of predicted metabolites | 148 | 226 | 327 | 465 |
| Number of successfully predicted reported metabolites^a^ | 33 | 41 | 52 | 60 |
| Number of molecules for which no metabolites could be predicted | 3 | 2 | 0 | 0 |
| Top-1 | 65.52 %^b^ | 65.52 % | 68.97 % | 68.97 % |
| Top-2 | 65.52 %^b^ | 65.52 % | 72.41 % | 72.41 % |
| Top-3 | 65.52 %^b^ | 68.97 % | 75.86 % | 75.86 % |

^a^ The total number of reported metabolites in the dataset was 81.

^b^ Note: If it existed, the best rank of the a known metabolite was always 1 for the SoM probability cutoff of 0.4. No known metabolite was predicted for nearly half of the parent molecules.

#

#

# References

1. FP Guengerich. Common and uncommon cytochrome P450 reactions related to metabolism and chemical toxicity. *Chem Res Toxicol* 14, 611–650 (2001)

2. PR Ortiz de Montellano; SD Nelson. Rearrangement reactions catalyzed by cytochrome P450s. *Arch Biochem Biophys* 507, 95–110 (2011)

3. PG Wislocki; GT Miwa; AΥH Lu. Reactions Catalyzed by the Cytochrome P-450 System. In: Enzymatic Basis of Detoxication. WB Jakoby, ed. , Elsevier (1980)

4. J Bolleddula; K DeMent; JP Driscoll; P Worboys; PJ Brassil; DL Bourdet. Biotransformation and bioactivation reactions of alicyclic amines in drug molecules. *Drug Metab Rev* 46, 379–419 (2014)

5. M Murray. Toxicological actions of plant-derived and anthropogenic methylenedioxyphenyl-substituted chemicals in mammals and insects. *J Toxicol Environ Health B Crit Rev* 15, 365–395 (2012)

6. PR Ortiz de Montellano. Substrate Oxidation by Cytochrome P450 Enzymes. In: Cytochrome P450: Structure, Mechanism, and Biochemistry. PR Ortiz de Montellano, ed. , Springer (2015)

7. B Testa; SD Krämer. The Biochemistry of Drug Metabolism – An Introduction Part 2. Redox Reactions and Their Enzymes. *Chem Biodivers* 4, 257–405 (2007)

8. A Parkinson. Biotransformation of Xenobiotics. In: Casarett and Doull’s Toxicology: The Basic Science of Poisons. CD Klaassen, ed. , McGraw-Hill (2007)

9. IM Rietjens; C den Besten; RP Hanzlik; PJ van Bladeren. Cytochrome P450-catalyzed oxidation of halobenzene derivatives. *Chem Res Toxicol* 10, 629–635 (1997)

10. S-F Zhou; Z-W Zhou; L-P Yang; J-P Cai. Substrates, inducers, inhibitors and structure-activity relationships of human Cytochrome P450 2C9 and implications in drug development. *Curr Med Chem* 16, 3480–3675 (2009)

11. K Auclair; Z Hu; DM Little; PRO de Montellano; JT Groves. Revisiting the Mechanism of P450 Enzymes with the Radical Clocks Norcarane and Spiro[2,5]octane. *J Am Chem Soc* 124, 6020–6027 (2002)
